# Glucocorticoid reduction after starting crinecerfont in pediatric patients with classic congenital adrenal hyperplasia: practical perspectives

**DOI:** 10.1210/clinem/dgag192

**Published:** 2026-05-04

**Authors:** Natalie J Nokoff, Patricia Y Fechner, Mimi S Kim, Ian Marshall, Deborah P Merke, Kyriakie Sarafoglou, Andrea L Hartzell, Vivian H Lin, Paul Thornton

**Affiliations:** Department of Pediatrics, University of Colorado Anschutz, Aurora, CO 80045, USA; Department of Pediatrics, University of Washington School of Medicine, Seattle Children's Hospital, Seattle, WA 98105, USA; Department of Pediatrics, Keck School of Medicine, University of Southern California, Children's Hospital Los Angeles, Los Angeles, CA 90027, USA; Department of Pediatrics, Rutgers-Robert Wood Johnson Medical School, New Brunswick, NJ 08901, USA; Department of Pediatrics, National Institutes of Health Clinical Center, Bethesda, MD 20892, USA; Eunice Kennedy Shriver National Institute of Child Health and Human Development, Bethesda, MD 20892, USA; Divisions of Pediatric Endocrinology and Genetics and Metabolism, University of Minnesota Medical School, Minneapolis, MN 55454, USA; Department of Experimental and Clinical Pharmacology, University of Minnesota College of Pharmacy, Minneapolis, MN 55455, USA; Medical Affairs, Neurocrine Biosciences, Inc., San Diego, CA 92130, USA; Medical Affairs, Neurocrine Biosciences, Inc., San Diego, CA 92130, USA; Division of Endocrinology, Cook Children's Medical Center, Fort Worth, TX 76104, USA; Department of Pediatrics, Burnett School of Medicine, Texas Christian University, Fort Worth, TX 76104, USA

**Keywords:** androstenedione, crinecerfont, congenital adrenal hyperplasia, pediatric, glucocorticoid

## Abstract

**Context:**

New and emerging non-glucocorticoid therapies for classic congenital adrenal hyperplasia (CAH) can reduce adrenocorticotropic hormone-mediated androgen production, allowing for glucocorticoid (GC) dose reductions. With the approval of crinecerfont as an adjunctive treatment to GC replacement for patients with classic CAH 4 years of age and older, expert recommendations were developed to provide guidance for GC reduction in pediatric patients after starting crinecerfont.

**Evidence Acquisition:**

In December 2024, 11 expert endocrinologists participated in a panel to provide input on strategies and considerations when reducing GC doses after introducing crinecerfont. A smaller panel reconvened in January 2025 to review previous discussions and develop recommendations for GC dose reduction after starting crinecerfont in pediatric patients with classic CAH (4-17 years).

**Evidence Synthesis:**

Approaches to GC reduction should be tailored to individual clinical goals, cortisol needs, and lifestyle. In pediatric patients, GC dose reductions should be guided by androgen concentrations, with the general goal of maintaining androgens near normal range to achieve normal growth and normalize bone age maturation while also minimizing complications from long-term GC exposure. Glucocorticoid doses should be reduced gradually with frequent monitoring and should not be decreased below the dose needed for physiologic cortisol replacement.

**Conclusion:**

The approval of crinecerfont has initiated a shift in the treatment approach for classic CAH, in which GCs are used at lower doses predominantly for cortisol replacement. These recommendations will become increasingly relevant as treatment for these patients continues to shift toward a new paradigm of physiologic GC replacement with adjunctive control of androgens.

## Introduction

Classic congenital adrenal hyperplasia (CAH), including both the salt-wasting and simple virilizing forms, is an autosomal recessive condition characterized by impaired synthesis of cortisol (1, 2). The most common type of CAH (∼95% of cases) is due to pathogenic variants in the *CYP21A2* gene encoding for 21-hydroxylase, an adrenal enzyme required for production of cortisol and aldosterone; consequently, most patients have both cortisol and aldosterone deficiencies (1-5). In addition, patients may also have epinephrine deficiency, especially those with the salt-wasting form (6, 7). Reduced production of cortisol results in increased secretion of corticotropin-releasing factor (CRF) and adrenocorticotropic hormone (ACTH), leading to increased stimulation of adrenal steroidogenesis. This results in excess production of adrenal androgens (hereafter referred to as “androgens”), as steroid precursors that accumulate proximal to the enzyme block are shunted down the androgen pathway (1, 2). The second most common enzyme deficiency (∼5% of cases), 11β-hydroxylase deficiency, shares several clinical features with 21-hydroxylase deficiency, including cortisol deficiency and excess androgen production. For the purpose of this manuscript, we will focus on classic 21-hydroxylase deficiency (hereafter referred to as “CAH”), as general principles of disease management also pertain to 11β-hydroxylase deficiency and other rare types of CAH.

Glucocorticoid (GC) treatment serves 2 distinct functions in patients with CAH: (1) replacing endogenous cortisol and (2) reducing ACTH-driven androgen production. When using GCs to reduce ACTH-driven androgen production, supraphysiologic GC doses (ie, doses higher than needed to treat adrenal insufficiency) are often needed (8-10); however, long-term supraphysiologic GC treatment can lead to multiple health comorbidities (2, 11). New, adjunctive, non-GC therapies for CAH reduce ACTH-mediated androgen production and can allow for reductions in GC doses to a more physiologic range. In light of the recent Food and Drug Administration (FDA) approval of the CRF type 1 receptor (CRF_1_) antagonist crinecerfont as an adjunctive treatment for classic CAH for patients 4 years of age and older (12), this manuscript aims to: (1) review disease-related consequences of CAH, (2) review existing and new treatments for CAH, and (3) provide recommendations from expert endocrinologists on how to approach GC dose reduction after starting crinecerfont in pediatric patients.

## Disease-related consequences of CAH

Patients with CAH have severe cortisol deficiency, and often aldosterone deficiency, and are at risk of a potentially life-threatening adrenal crisis (13). Infants and young children are particularly prone to hypoglycemia due to cortisol and epinephrine deficiency (14), compounded by lower glycogen stores (15). Approximately 8% of patients with CAH experience hypoglycemia early in life; hypoglycemia can occur at any age during an adrenal crisis (16-19).

Exposure to excess ACTH and/or androgens affects individuals with CAH across all stages of life (11, 20), including: prenatal genital virilization in 46,XX infants and postnatal clitoromegaly (20-22); premature pubarche, accelerated growth, advanced epiphyseal maturation, and central precocious puberty in growing children (23-28); and hirsutism, acne, and oligomenorrhea in adolescent girls (16, 22, 29, 30). Poor disease control with elevated ACTH can result in testicular adrenal rest tumors in male patients (1, 2, 31), which typically develop during adolescence (median age of onset: 13.6 years (32)) and are the most common cause of male infertility in CAH. Furthermore, the negative feedback from excess peripheral androgens can lead to hypogonadotropic hypogonadism (16, 33-36). Finally, androgen excess may play a role in the development of cardiometabolic risk factors (37-40).

## GC treatment and adverse effects in pediatric patients with CAH

Historically, treatment of CAH with GCs has been a difficult balance between managing hyperandrogenism and avoiding hypercortisolism (1). The Endocrine Society 2016 practice guideline for the treatment of primary adrenal insufficiency recommends ∼8 mg/m^2^/day of hydrocortisone for children (8). In contrast, the 2018 practice guideline for CAH recommends 10 to 15 mg/m^2^/day for pediatric patients for adequate androgen control, underscoring that supraphysiologic GC doses are often needed when managing androgens with GCs in the absence of adjunct therapies (1). For patients with CAH who are still growing, hydrocortisone is the preferred GC therapy because its short half-life reduces the adverse effects associated with longer-acting, more potent GCs, particularly growth suppression (10, 11, 16, 20). Many pediatric endocrinologists give hydrocortisone in 3 divided doses as recommended in guidelines (1, 41). Some clinicians prefer dosing 4 times per day to minimize periods of hypocortisolemia, as supported by cortisol pharmacokinetic modeling studies (42, 43), but this is not always feasible or practical for patients and their families. Some physicians employ a circadian dosing schedule (ie, highest GC dose in the morning, as would be typical for primary adrenal insufficiency), though some prefer a reverse-circadian dosing regimen (ie, higher GC dose in the evening/bedtime) to attenuate the early morning rise in androgens. A cross-over study comparing the 2 approaches showed that administration of the highest hydrocortisone dose in the evening resulted in significantly lower 17-hydroxyprogesterone (17-OHP) at 5 Am, whereas administering the highest dose in the morning resulted in lower 17-OHP and androstenedione in the afternoon (44). However, evidence for the benefits of reverse-circadian dosing is limited, and high nocturnal cortisol may affect sleep quality, reduce growth hormone secretion, and have deleterious metabolic effects (10, 45, 46).

Real-world studies have reported wide variability in GC regimens, with GC doses that are often above the recommended range (27, 47-52). Prolonged exposure to supraphysiologic GCs has been associated with well-documented adverse health effects (2, 3, 11, 27, 39, 49, 53-71). In growing patients, GCs can exert growth-suppressing effects by interfering with growth hormone secretion, with higher doses associated with shorter predicted adult height (27, 54, 58, 59, 62, 63). A longitudinal study of children with CAH found that adult height was decreased by 0.37 cm for each 1 mg/m^2^/day increase in hydrocortisone dose (27). Moreover, long-term supraphysiologic GC use can adversely impact bone health starting in childhood. Studies in children and adolescents with CAH have shown that higher GC doses were negatively correlated with bone mineral density and with bone mineral content at the femur and the spine (64, 65), increasing the risk of osteoporosis and fractures in adulthood (3, 53, 55, 56, 66-69).

Even before reaching adulthood, children and adolescents with CAH have an increased risk for developing multiple cardiometabolic complications, including obesity, hypertension, hyperglycemia, insulin resistance, and hypercholesteremia, in part as a result of chronic supraphysiologic GC exposure (10, 39, 49, 56-58, 60, 61, 70-75). Obesity has been reported to occur in up to 43% of pediatric patients with CAH compared with ∼20% in the general population (57, 58). Youth with CAH have 3.2× greater odds of overweight/obesity than matched controls (76) and have earlier adiposity rebound (28, 57, 77). In addition, children with CAH have a higher fat mass-to-lean mass ratio and more centralized fat distribution than control participants (39, 49, 70). Pediatric patients with CAH also have a higher prevalence of elevated fasting glucose (72) and increased insulin resistance than their peers (even after adjusting for body mass index) (70-72). Insulin resistance has been positively correlated with higher GC dosing (70, 74).

Finally, delayed diagnosis, poor disease control, hyponatremic episodes, supraphysiologic GC exposure, and androgen excess have been associated with decreased quality of life and higher risk of neurocognitive and mental health problems (56, 78-87). Children with CAH are at increased risk of developmental delay and difficulty with executive function and working memory (88-90). Patients with CAH also have an increased prevalence of depressive disorders, anxiety disorders, and attention deficit hyperactivity disorder (78, 79, 85-87, 91). Furthermore, poor disease control has been independently associated with behavioral problems (86).

## New approaches to managing CAH

There are several unmet needs in the management of CAH warranting new approaches to treatment (92). Pediatric patients need approaches that allow for lower androgens and more physiologic GC treatment, improved linear growth, and reduced risk of premature pubarche, precocious puberty, obesity, and cardiometabolic disease.

Emerging GC therapies have aimed to allow for more accurate GC dose administration with the addition of new formulations of hydrocortisone including hydrocortisone oral granules (Alkindi Sprinkle^®^) and oral solution (Khindivi^®^ [FDA-approved for patients with adrenal insufficiency ≥5 years of age]) (92). While both Alkindi Sprinkle and Khindivi provide promising options for more accurate administration, it is important to note that Khindivi contains inactive ingredients (polyethylene glycol 400, propylene glycol, and glycerin) that may cause systemic adverse reactions in some patients, and the excipients can cause toxicity in young children due to hyperosmolarity (93).

Additional therapies aim to better mimic the natural circadian rhythm of cortisol with improved GC delivery (92). One approach is through the use of continuous subcutaneous hydrocortisone infusion (unlicensed) (94, 95), with recently completed phase 1 studies in children with CAH reporting improvements in androgen control after 14 weeks (96). Another approach is through modified-release preparations of hydrocortisone. Efmody^®^ is a twice-daily modified-release hydrocortisone designed to approximate the physiologic diurnal cortisol rhythm (licensed in Europe) (97). Plenadren^®^, a once-daily dual-release oral hydrocortisone with an extended-release core and an immediate-release coat designed to be taken upon awakening, provides a near-physiologic cortisol profile early in the day but lacks nighttime coverage and has not been studied in children (licensed in Europe for adults with adrenal insufficiency, with an ongoing trial in adults with CAH [NCT03760835]).

In addition, several new non-GC approaches are approved or in development to reduce ACTH-mediated androgen production when added as an adjunctive treatment to the patient's usual GC regimen (92). These adjunctive treatment strategies would not eliminate the need for physiologic cortisol replacement but allow for those on supraphysiologic GC regimens to achieve lower, more physiologic daily doses. Therapies currently under investigation include a melanocortin type 2 receptor antagonist (atumelnant [NCT05907291, NCT07144163, NCT07159841, NCT06712823] (98)) and an anti-ACTH monoclonal antibody (asedebart, previously Lu AG13909 [NCT05669950] (99)), although no data have been reported on their use in pediatric patients to date.

**Figure 1:**
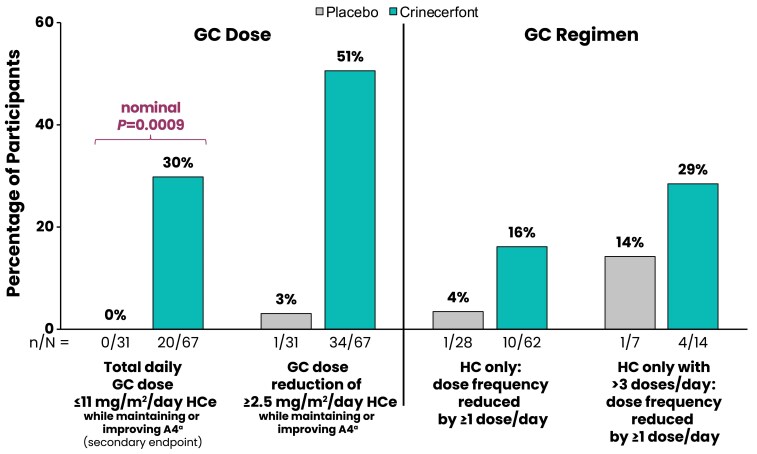
Achievement of reductions in GC dose or GC regimen response threshold at the end of double-blind placebo-controlled treatment in the phase 3 CAHtalyst^®^ Pediatric trial of crinecerfont (104, 105). ^a^ Maintaining or improving A4 relative to baseline was defined as A4 ≤120% of baseline or ≤ULN according to sex and either age (for Tanner stage 1) or pubertal stage (for Tanner stages 2-5) for the CAHtalyst Pediatric trial. Abbreviations: A4, androstenedione; GC, glucocorticoid; HC, hydrocortisone; HCe, hydrocortisone equivalents.

Crinecerfont is a first-in-class oral CRF_1_ antagonist that is FDA-approved in patients with classic CAH 4 years of age and older as an adjunctive treatment to the GC replacement required to treat the underlying adrenal insufficiency (12). By reducing elevated ACTH at the level of the pituitary, crinecerfont controls androgens in both the salt-wasting and simple virilizing forms of CAH. In clinical trials of crinecerfont in pediatric and adult patients with CAH, substantial decreases in ACTH, 17-OHP, and androstenedione were observed when participants were treated with crinecerfont in addition to their usual GC regimen (100-104). In the phase 3 studies, these decreases enabled clinically meaningful reductions in GC dose with crinecerfont compared with placebo while maintaining or improving androstenedione relative to baseline (Fig. 1) (102-104, 106). With the availability of an adjunctive treatment to control androgen production, GC doses can be reduced toward physiologic replacement needs, potentially reducing the negative effects of chronic supraphysiologic GC treatment.

## Development of recommendations for GC dose reductions after initiating crinecerfont

In December 2024, 11 endocrinologists (6 pediatric, 5 adult) with extensive experience treating and managing patients with CAH were invited by Neurocrine Biosciences, Inc. (San Diego, CA) to participate in an expert panel on GC dose reduction after introducing crinecerfont into the treatment paradigm. These experts provided input on the overall strategy and key considerations when reducing GC doses in patients with CAH after initiating crinecerfont treatment. A smaller group of 5 expert endocrinologists (3 pediatric, 2 adult) reconvened in January 2025 to review the previous discussions and develop recommendations for pediatric and adult patients, respectively. All authors reviewed the recommendations, provided feedback, and contributed to the final algorithm and manuscript. Here, we report the recommendations for reducing GC doses after starting crinecerfont in pediatric patients with CAH; recommendations for adult patients are reported in a companion manuscript (107).

## Practical perspectives and recommendations

These recommendations are intended to serve as general guidance for reducing GC doses after starting crinecerfont in pediatric patients with CAH; however, they may not be appropriate for every patient. Adjustments to GC treatment should be individualized based on each patient's cortisol needs and clinical goals with respect to achieving appropriate androgen control, while avoiding complications of both GC excess and adrenal insufficiency. All treatment adjustments should be based on clinician experience and shared decision-making with patients and their parents/caregivers.

### Initial assessments and starting crinecerfont

Prior to initiation, it is important to discuss the expectations, goals, and rationale for starting crinecerfont with patients and families. Treatment decisions regarding starting crinecerfont and potential GC dose reductions should be individualized based on each patient's goals and the expertise of their treating provider. Crinecerfont treatment may benefit a wide range of patients with varying clinical goals, including managing ACTH-driven androgens, reducing GC dose, changing GC type, and/or simplifying the daily GC regimen. Growing patients with CAH may prioritize reducing both androgens and GC doses to optimize growth and development. During puberty and adolescence, reducing ACTH-driven androgens may help achieve normal pubertal development, minimize clinical signs of hyperandrogenism such as hirsutism and irregular menses in female patients, and might reduce the risk of worsening testicular adrenal rest tumors in male patients. In addition, given the well-documented negative effects of long-term excess GC exposure, many patients may benefit from reducing their GC dose, especially those with comorbidities impacted by hypercortisolism (eg, weight gain/obesity, insulin resistance) and/or suppressed androstenedione.

Before starting crinecerfont, it is also critical to emphasize to the patient and their parents/caregivers the risk of life-threatening adrenal crisis if they stop taking their GC medication (see Adrenal crisis). While adjunctive non-GC therapies such as crinecerfont can reduce excess ACTH and/or androgens, thereby allowing for lower GC doses, GC treatment is still required for endogenous cortisol replacement and should never be discontinued in patients with CAH. In addition, patients and parents/caregivers should be educated on stress dosing (see Stress dosing) and the need to monitor for symptoms of adrenal insufficiency (see Adrenal insufficiency), including mineralocorticoid insufficiency. Patients should also be counseled about the symptoms of GC withdrawal (see GC withdrawal), which can occur in any individual undergoing dose reductions but may be more common in those previously treated with higher GC doses or longer-acting GCs (108).

At the initial visit, patients and their parents/caregivers should be informed about relevant prescribing information for crinecerfont, including the available formulations (oral solution and soft gelatin capsule) and weight-based dosing recommendations for growing pediatric patients (25 mg twice daily for patients with a weight of 10 to <20 kg; 50 mg twice daily for 20 to <55 kg; or 100 mg twice daily for ≥55 kg) (12). In addition, it is important to advise the patient to take crinecerfont with a snack or meal with at least 5 g of fat (eg, 8 oz of 2% milk) for sufficient absorption. Like with many medications, consistency is important. Commonly reported adverse reactions with crinecerfont in pediatric patients include headache, abdominal pain, fatigue, nasal congestion, and epistaxis (Table 1). Patients taking concomitant strong or moderate cytochrome P450 3A4 (CYP3A4) inducers should increase their crinecerfont dose following the dosage modifications outlined in the US Prescribing Information (12). In general, drug–drug interactions should always be considered for concomitant medications used to treat chronic conditions.

**Table 1:** Commonly reported adverse reactions with crinecerfont in pediatric patients with classic CAH

Common adverse reactions, *n* (%)*^a^*	Crinecerfont (*N* = 69)	Placebo (*N* = 33)
Headache	17 (25)	2 (6)
Abdominal pain*^b^*	9 (13)	0
Fatigue	5 (7)	0
Nasal congestion	5 (7)	1 (3)
Epistaxis	3 (4)	0

Abbreviations: CAH, congenital adrenal hyperplasia; MedDRA, Medical Dictionary for Regulatory Activities.

^*a*^Defined in the US Prescribing Information of crinecerfont as occurring at an incidence of at least 4% and greater than placebo during the double-blind placebo-controlled period of the phase 3 CAHtalyst Pediatric trial (12).

^*b*^Includes the following MedDRA preferred terms: abdominal pain, upper abdominal pain, and lower abdominal pain.

**Figure 2:**
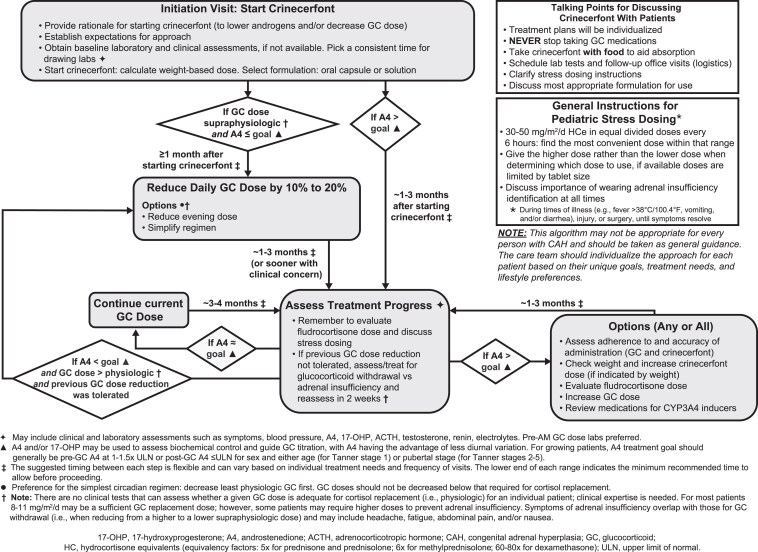
Approach to GC reduction with initiation of crinecerfont in pediatric patients with classic CAH (4-17 years): practical perspectives (107).

Before starting crinecerfont, baseline laboratory and clinical assessments should be obtained if they are not currently available, including assessment of androgen control (may include androstenedione, 17-OHP, ACTH, and testosterone) and mineralocorticoid replacement (renin and electrolytes as needed, Fig. 2). The Endocrine Society guidelines do not specify when to measure labs but recommend consistency in measurements with regard to the time of day (eg, morning or afternoon) and medication administration (eg, before or after taking GC medications) (1) as there can be a wide variability (>60% change) in 17-OHP and androstenedione concentrations even within a 2-hour time frame from administration of the GC dose (10). As the rise of androgens in the early morning hours is one of the biggest hurdles in managing CAH, especially in children, many clinicians may prefer measuring androgens before the morning GC dose in order to assess underlying androgen concentrations without the confounding factor of a recent GC dose. Conversely, some clinicians measure androgens 2 to 4 hours after the GC dose to assess maximal androgen response (43), while others measure 6 to 7 hours after the morning GC dose to evaluate whether the morning dose provides adequate control before the midday dose (10). In addition, practical reasons, such as time of appointment, family schedule, or laboratory operating hours, may dictate the timing of the lab measurements. Consistency between laboratory measurements is recommended, as well as documentation of timing of the labs relative to the last GC dose.

In addition to labs, patients should also be regularly monitored for changes in growth velocity, poor adherence, signs of hyperandrogenism (eg, advanced bone age, acne, and hirsutism), hyperpigmentation, and signs of hypercortisolism (eg, Cushingoid features), indicating either excess androgen and/or GC exposure, as these findings may impact decision-making for the clinician.

### Setting a goal GC dose

Even as novel non-GC adjunctive therapies may mitigate the need for supraphysiologic doses, GC doses must not be reduced below that required for cortisol replacement. There is no currently available clinical test that can determine physiologic dose requirements; however, studies of daily cortisol production rates in healthy individuals can help to inform what may be a physiologic GC replacement dose for pediatric patients with CAH. In a study conducted in healthy children and adolescents (8-17 years), the mean daily cortisol production rate was ∼7 mg/m^2^/day, and a few patients had a daily cortisol production rate as high as 11 mg/m^2^/day; adults have a similar mean with a range up to 14 mg/m^2^/day (109, 110). In a trial of 62 prepubertal children with CAH, those randomized to a reduced GC dose (with an antiandrogen and aromatase inhibitor) were able to maintain a hydrocortisone dose of 8 mg/m^2^/day with no increase in adrenal crisis rates compared with control participants (on 15 mg/m^2^/day) (111). Furthermore, the crinecerfont phase 3 pediatric study in CAH aimed to reduce the GC dose to 8 to 10 mg/m^2^/day, and no adrenal crisis events were observed during the 6-month double-blind period (104). Taken together, these studies indicate that, while 8 to 11 mg/m^2^/day may be a sufficient physiologic GC replacement dose for most patients, there is no single cutoff for a physiologic GC dose and some patients may require higher doses to prevent adrenal insufficiency. Treatment decisions regarding a target GC dose must be individualized for each patient using shared decision-making and clinician expertise (11).

### Approach to GC dose reductions after initiating crinecerfont

Target ranges for androgens may vary between patients and depend on age, sex, pubertal stage, timing of laboratory assessments, and individual treatment goals. Androstenedione and 17-OHP may be used to assess biochemical control and guide GC titration, with androstenedione having the advantage of less diurnal variation (1, 10). For androstenedione, a general goal may be 1-1.5× the upper limit of the normal range based on age, sex, and pubertal stage when measured prior to the morning GC dose or within the normal range when measured after the morning GC dose. For 17-OHP, serum levels of ≤1200 ng/dL may be considered an acceptable target for many patients as levels within the normal range may reflect excess GC exposure (1, 10, 112).

#### If baseline androgens are at or below goal and on a supraphysiologic GC dose

If the baseline androgens are at or below goal and the patient is receiving a supraphysiologic GC dose, GC reductions can be initiated as early as 1 month after starting crinecerfont. The total daily GC dose may be reduced by 10% to 20% at a time in a stepwise manner (Fig. 2). The degree of GC dose reduction should be individualized based on the patient's treatment goals and what is practical (eg, available tablet dose sizes) and should not be reduced below the dose needed for cortisol replacement. When reducing GC doses for hydrocortisone tablets, it is generally recommended to avoid selecting a dose that would require splitting tablets beyond half a tablet, as this may lead to inaccurate dosing (113, 114). If tablet splitting is not feasible for the target GC dose, different hydrocortisone formulations (eg, oral granules [Alkindi Sprinkle], oral solution [Khindivi, for those ≥5 years of age]) may be considered. While use of dexamethasone is uncommon in growing pediatric patients, if a patient is on dexamethasone, consider switching their GC regimen from dexamethasone to another GC (eg, prednisolone) prior to reducing GC dose. Note that for GC replacement, the hydrocortisone equivalency factors for dexamethasone and prednisolone are 60 to 80× and 5×, respectively; consequently, 0.5 mg dexamethasone ≈6 to 8 mg prednisolone ≈30 to 40 mg hydrocortisone (58, 108, 115-117).

As a first step, consider reducing the GC dose that is given at the most nonphysiologic time (ie, bedtime or evening dose), as this may better mimic the natural circadian rhythm of cortisol secretion (118, 119). The therapeutic dose regimen of crinecerfont is expected to provide plasma exposures that ensure continuous CRF_1_ inhibition throughout the full day. It may also be possible to simplify the GC regimen by consolidating doses. For example, if a patient is on 2 morning doses of hydrocortisone (eg, a patient is on both a 5 Am dose and a 7 Am dose), it may be possible to consolidate these into a single, reduced morning dose. In growing patients, a hydrocortisone dosing frequency of at least 3 doses per day is preferred to avoid periods of hypercortisolemia and hypocortisolemia throughout the day (1, 43, 117, 120, 121). As well, it may be appropriate to allow patients to “outgrow” their GC dose rather than reduce their current GC dose, as physiologic cortisol needs typically increase over time, particularly during puberty when cortisol clearance is increased (122).

Following all GC dose reductions, it is important to assess androgen concentrations and monitor for signs of hyperandrogenism. Growth velocity and bone age maturation should also be monitored in growing children, as recommended in guidelines. As with all medications, adherence should be discussed.

All patients should also be monitored for signs of mineralocorticoid insufficiency or excess. Reducing the daily hydrocortisone dose can increase the risk of mineralocorticoid insufficiency as the mineralocorticoid effect of hydrocortisone is decreased. On the other hand, in vitro studies have shown that very high 17-OHP concentrations have anti-mineralocorticoid activity (123). Consequently, as disease control improves and 17-OHP decreases, the risk of salt-wasting may lessen. Monitoring of plasma renin activity, electrolytes, and blood pressure should be performed during clinic visits with appropriate dose adjustments of fludrocortisone, if needed. If there are signs of mineralocorticoid insufficiency (see Adrenal insufficiency), consider increasing the fludrocortisone dose. Elevated blood pressure and suppressed plasma renin activity can indicate mineralocorticoid excess (60, 124, 125), and a reduction in fludrocortisone dose should be considered. Finally, it is essential to monitor for signs of adrenal insufficiency and GC withdrawal when reducing GC doses (see Adrenal insufficiency and GC withdrawal).

Approximately 1 to 3 months after the initial GC dose reduction, assess treatment progress and evaluate whether further GC dose reduction is appropriate (Fig. 2). Intervals between GC reduction steps are flexible and may vary based on the patient's goals as well as external factors, such as the frequency of office visits and/or the time to obtain results from laboratory assessments. *If androgens are below the goal for the patient and the previous GC dose reduction was well tolerated*, consider further reducing the GC dose while continuing to monitor androgens and for signs of adrenal insufficiency, GC withdrawal, and mineralocorticoid insufficiency/excess. *If androgens are at or near goal*, continue at the same GC dose and reevaluate treatment progress at the next routine clinic visit. *If androgens are above goal*, pause GC reductions and consider any or all of the following options as next steps: (1) ensure lab assessments being compared were obtained at approximately the same time of day and the same time relative to the last GC dose; (2) assess adherence to and accuracy of administration of crinecerfont (in particular, whether the patient is taking it with food or drink that contains some fat to aid absorption) and other CAH medications—this can be particularly important in adolescents who may become less adherent as they gain increasing independence in self-managing their care; (3) assess the patient's weight to determine whether their crinecerfont dose needs to be increased; (4) assess the adequacy of the patient's mineralocorticoid replacement and salt and water balance; (5) evaluate whether the patient has started any new medications that are CYP3A4 inducers; and/or (6) consider increasing the GC dose. In all scenarios, continue to conduct clinical and laboratory assessments at routine clinic visits to monitor treatment progress and adjust treatment as needed.

#### If baseline androgens are above goal

For patients whose baseline androgens are above goal (regardless of GC dose) prior to starting crinecerfont, GC doses should be kept stable for ∼1 to 3 months after crinecerfont initiation before assessing treatment progress (Fig. 2). Androgens can be assessed as soon as 1 month after starting crinecerfont if needed to inform reduction in GC dosage; however, the length of time needed for androgens to reach goal after starting crinecerfont may vary for each patient, and the decision to begin GC reductions should be individually determined based on the patient's goals and clinician judgment. For example, if the patient's primary goal is androgen reduction and GC exposure is not a clinical concern, it may be appropriate to consider keeping GC doses stable for a longer period than 3 months (or indefinitely). At the next clinic visit, assess the patient's treatment progress and determine next steps based on androgens as described in the previous section.

### GC withdrawal

Glucocorticoid withdrawal can occur in any patient lowering their GC dose, but it is more likely to occur in patients on higher GC doses, patients taking long-acting GCs (eg, prednisone and dexamethasone), or in patients whose GC dose is reduced quickly (108). Symptoms of GC withdrawal are summarized in Table 2 and may be nonspecific (126). If GC withdrawal symptoms occur, they should be resolved for at least 2 weeks before attempting another GC dose reduction. Patients who experience intolerable GC withdrawal symptoms should increase at least halfway back to the prior GC dose and may attempt a smaller GC reduction ∼2 to 4 weeks after the symptoms resolve.

**Table 2:** Clinical features of adrenal insufficiency, adrenal crisis, and GC withdrawal

	Adrenal insufficiency		Adrenal crisis	GC withdrawal
	*Cortisol/GC insufficiency*	*Aldosterone/MC insufficiency*		
Signs and symptoms	Fatigue, nausea, muscle pain, joint pain, headache, general malaise, unintentional weight loss, orthostatic hypotension, tachycardia, abdominal ache, dizziness	Salt craving, anorexia, muscle weakness, fatigue, dizziness, orthostatic hypotension	Hypovolemic shock, hypotension, nausea or vomiting, fever, impaired consciousness	Fatigue, nausea, muscle pain, joint pain, headache, general malaise, sleep disturbances, mood change
Laboratory abnormalities	Hyponatremia, hypoglycemia, elevated ACTH	Hyperkalemia, hyponatremia, elevated PRA	Hyponatremia, hyperkalemia, elevated BUN, elevated creatinine, hypoglycemia, eosinophilia, lymphocytosis	
Timing of symptoms	Occurs when there is inadequate GC for cortisol replacement	Occurs when there is inadequate MC replacement and/or inadequate salt intake	Occurs during times of significant stress, especially infectious illnesses, with inadequate cortisol replacement	Occurs following GC dose reduction but dose remains physiologic or more. Can occur at any point during GC dose reduction, but is more likely in patients starting at higher GC doses
Treatment or resolution	Increase GC dose to previously tolerated dose	Consider increasing fludrocortisone dose and/or salt intake	Stress dosing (see Table 3) Hospital admission may be required based on severity of illness and symptoms	Pause GC dose reduction; if symptoms do not resolve, consider increasing GC dose and slowing the rate of further reductions

Abbreviations: ACTH, adrenocorticotropic hormone; BUN, blood urea nitrogen; GC, glucocorticoid, MC, mineralocorticoid; PRA, plasma renin activity.

### Adrenal insufficiency

#### Cortisol deficiency

Symptoms of cortisol insufficiency can be similar to those of GC withdrawal, may occur when GC doses fall below replacement needs, and will not resolve unless the GC dose is increased to provide sufficient cortisol replacement (Table 2).

#### Mineralocorticoid insufficiency

Mineralocorticoid insufficiency occurs when there is inadequate fludrocortisone replacement and/or salt intake and should be considered in patients experiencing symptoms of adrenal insufficiency, especially if there has been a change in lifestyle potentially exacerbating salt loss (ie, hot weather exposure, increase in sports activities; Table 2). Notably, some GCs (such as hydrocortisone) also have mineralocorticoid activity; thus, GC dose reduction requires reassessment of the adequacy of mineralocorticoid replacement. Dehydration can result in increased ACTH via a CRF_1_-independent mechanism by stimulation of pituitary vasopressin V1b receptors (127), so maintaining adequate hydration, salt, and mineralocorticoid replacement is important for maintaining disease control.

### Stress dosing

During times of significant physical stressors, GC stress dosing is required to prevent adrenal crisis (9, 19). It is common practice to recommend that patients triple their usual GC dose for their stress dose (9); however, this guidance may not be sufficient for patients who are taking reduced GC doses with crinecerfont. Our recommendations for stress dosing are provided in Table 3 and are consistent with consensus guidelines (1). Hydrocortisone is the preferred GC to use for stress dosing. If unavailable, other GCs may be used; however, dexamethasone is not recommended due to its slow onset of action and lack of mineralocorticoid activity. Hydrocortisone oral solution (Khindivi) is not approved for increased dosing during stressful or emergency situations due to greater exposure to inactive ingredients that may increase the risk of adverse reactions; therefore, patients should switch to a different hydrocortisone formulation when stress dosing is required.

**Table 3:** Stress dosing recommendations for pediatric patients with classic CAH

Situation	Recommendation
Competitive sports or physical exercise beyond usual routine	Extra 2.5-5 mg/m^2^/day HCe may be given on an individual basis
Emotional or mental stress	No increased dose required
Extreme emotional trauma (eg, death of a family member)	30-50 mg/m^2^/day HCe in equal divided doses every 6 hours
Any illness involving vomiting and/or diarrhea, without hemodynamic instability	50 mg/m^2^/day HCe in equal divided doses every 6 hours Repeat GC stress dose if vomiting occurs within 1 hour of oral administration Hydrate with electrolytes and include some glucose-containing fluids (eg, juice and soda) If unable to keep GC medication down (eg, repetitive vomiting, loss of consciousness, or other reason oral medication cannot be taken), use emergency hydrocortisone injection kit and visit the nearest emergency department
Fever >38°C/100.4°F	30-50 mg/m^2^/day HCe in equal divided doses every 6 hours Ingest simple and complex carbohydrates (15 g or one-half cup juice or regular soda) and fluids containing electrolytes regularly and increase fluid intake for dark urine
Fever > 39°C/102°F or severe infection (eg, pneumonia)	50 mg/m^2^/day HCe in equal divided doses every 6 hours Ingest simple and complex carbohydrates (15 g or one-half cup juice or regular soda) and fluids containing electrolytes regularly and increase fluid intake for dark urine ER visit and/or hospital admission may be required
Acute trauma (eg, fracture)	30-50 mg/m^2^/day HCe in equal divided doses every 6 hours
Minor procedure with no sedation	No dose increase needed
Short surgeries	Hydrocortisone 50 mg/m^2^ IM/IV bolus 30 minutes prior to general anesthesia
Major surgery	Hydrocortisone 50 mg/m^2^ IM/IV bolus followed either by Hydrocortisone 100 mg/m^2^/day by continuous infusion or 100 mg/m^2^/day IV in equal divided doses every 6 hours

Hydrocortisone is the preferred GC to use for stress dosing. If hydrocortisone is not available, other GCs can be used with the following conversion factors for HCe: prednisone and prednisolone (5×), methylprednisolone (6×). Dexamethasone is not appropriate for stress dosing for moderate and major stress due to its slow onset of action and lack of mineralocorticoid activity. Supportive care (eg, isotonic fluids, electrolytes, and/or glucose) should be provided as appropriate in situations of moderate and major stress. When the situation of stress has resolved and the patient is well, patients may resume their usual GC dose or taper the GC dose depending on the duration of stress dosing.

Abbreviations: CAH, congenital adrenal hyperplasia; GC, glucocorticoid; HCe, hydrocortisone equivalents.

### Adrenal crisis

Adrenal insufficiency can progress to an acute life-threatening adrenal crisis, especially during times of infectious illnesses (Table 2) (19, 128). Patients with salt-wasting CAH and those on lower GC doses are at highest risk of adrenal crisis (13, 19, 128). Glucocorticoid doses should not be reduced below the threshold needed for physiologic GC replacement while on crinecerfont, and stress dosing should be initiated at the first signs of illness.

Appropriate management of adrenal crisis requires immediate recognition of the clinical signs and symptoms of adrenal insufficiency, as well as knowledge of the situational circumstances that may trigger adrenal crisis (9). Early recognition and prompt intervention with stress dosing are critical to prevent long-lasting complications or death. Adolescent patients, parents, and care providers must be familiar with the administration of stress doses and the importance of hydration, electrolyte supplementation, and frequent carbohydrate intake. The family should be provided with a written Adrenal Insufficiency Action Plan and/or Emergency Care Letter to provide to emergency medical service providers, and patients should have age-appropriate emergency medical identification devices.

## Conclusions

Balancing the consequences of androgen excess with those of long-term supraphysiologic GC exposure has been an ongoing challenge in managing CAH. With the FDA approval of crinecerfont, the first non-GC adjunctive therapy to control androgens in patients with classic CAH, it is now possible for patients to reduce GCs to lower, more physiologic doses, potentially reducing the clinical complications associated with supraphysiologic GC treatment and excess androgens. In this article, we summarize approaches to GC dose reduction after starting crinecerfont in real-world clinical practice. We recommend that the approach be tailored to the individual patient and/or family's clinical goals, cortisol needs, and lifestyle/schedule. In pediatric patients, GC dose reductions should be guided by biomarkers of androgen excess and clinical status, with the goal of maintaining androgens near the normal range to achieve normal growth velocity and growth plate maturation, and prevent the development of long-term cardiometabolic, bone, and fertility complications. Glucocorticoid doses should be reduced gradually with frequent monitoring to minimize the risks of adrenal insufficiency/adrenal crisis and symptoms of GC withdrawal. Moreover, GC dose should not be decreased below that needed for physiologic cortisol replacement, and it is essential to educate patients and their parents/caregivers on stress dosing guidance to prevent life-threatening adrenal crisis.

The approval of crinecerfont has initiated a shift in the treatment approach for classic CAH, in which GCs can be used at lower, more physiologic doses with adjunctive management of androgens.

## Data Availability

Data sharing is not applicable to this article as no datasets were generated or analyzed. N. J. N. is an Editorial Board Member of the *Journal of Clinical Endocrinology & Metabolism* and played no role in the journal's evaluation of the manuscript.

## Abbreviations

17-OHP: 17-hydroxyprogesterone
ACTH: adrenocorticotropic hormone
CAH: congenital adrenal hyperplasia
FDA: Food and Drug Administration
GC: glucocorticoid
CRF: corticotropin-releasing factor
CRF_1_: CRF type 1 receptor
